# Predicting mental and psychomotor delay in very pre-term infants using machine learning

**DOI:** 10.1038/s41390-023-02713-z

**Published:** 2023-07-27

**Authors:** Gözde M. Demirci, Phyllis M. Kittler, Ha T. T. Phan, Anne D. Gordon, Michael J. Flory, Santosh M. Parab, Chia-Ling Tsai

**Affiliations:** 1 Computer Science Department, The Graduate Center of the City University of NY, New York, NY USA; 2 Department of Infant Development, NYS Institute for Basic Research in Developmental Disabilities, Staten Island, NY USA; 3 Pediatrics, Richmond University Medical Center, Staten Island, NY USA; 4 Computer Science Department, Queens College of the City University of NY, Flushing, NY USA

## Abstract

**Background:**

Very preterm infants are at elevated risk for neurodevelopmental delays. Earlier prediction of delays allows timelier intervention and improved outcomes. Machine learning (ML) was used to predict mental and psychomotor delay at 25 months.

**Methods:**

We applied RandomForest classifier to data from 1109 very preterm infants recruited over 20 years. ML selected key predictors from 52 perinatal and 16 longitudinal variables (1–22 mo assessments). SHapley Additive exPlanations provided model interpretability.

**Results:**

Balanced accuracy with perinatal variables was 62%/61% (mental/psychomotor). Top predictors of mental and psychomotor delay overlapped and included: birth year, days in hospital, antenatal MgSO_4,_ days intubated, birth weight, abnormal cranial ultrasound, gestational age, mom’s age and education, and intrauterine growth restriction. Highest balanced accuracy was achieved with 19-month follow-up scores and perinatal variables (72%/73%).

**Conclusions:**

Combining perinatal and longitudinal data, ML modeling predicted 24 month mental/psychomotor delay in very preterm infants ½ year early, allowing intervention to start that much sooner. Modeling using only perinatal features fell short of clinical application. Birth year’s importance reflected a linear decline in predicting delay as birth year became more recent.

**Impact:**

- Combining perinatal and longitudinal data, ML modeling was able to predict 24 month mental/psychomotor delay in very preterm infants ½ year early (25% of their lives) potentially advancing implementation of intervention services.
- Although cognitive/verbal and fine/gross motor delays require separate interventions, in very preterm infants there is substantial overlap in the risk factors that can be used to predict these delays.
- Birth year has an important effect on ML prediction of delay in very preterm infants, with those born more recently (1989–2009) being increasing less likely to be delayed, perhaps reflecting advances in medical practice.

## Introduction

Current worldwide estimates are that 1 in 10 infants are born preterm and that approximately 1 million die annually as a result of preterm birth.^1^ There has been increasing success at keeping the very youngest of these infants alive, but they are at heightened risk for neurodevelopmental delays.^2–4^ Given the emotional investment of families and ongoing costs to society, research in preterm infants’ subsequent development has been intense. Understanding effects of biological, medical and environmental risks potentially improves decision making, treatment and early intervention, such as cognitive, communication, and motor therapies that have been provided since before the U.S.A’s Individuals with Disabilities Education Act. Earlier identification of infants likely to be delayed is critical to providing intervention that can maximize children’s potentials.^5–7^ Toward this end, a large body of research aims to connect risk factors with mortality/morbidity and to predict who will be delayed. To date, mostly standard regression or multivariate prediction have been used (for reviews see^8,9^). Crilly et al.^8^ suggest that prediction research might benefit from non-linear tools such as machine learning (ML).

Recent studies of medical outcomes in preterm infants have begun to utilize ML. Podda et al.^10^ utilized Artificial Neural Networks to predict in-hospital mortality among pre-term infants *<*30 weeks gestational age (GA) and reported their predictor had “slightly better” discrimination than achieved with logistic regression. Similarly, Feng et al.^11^ developed a deep learning model to predict mortality among preterm infants employing dynamically sampled vital signs in conjunction with static clinical variables. Also predicting mortality, Lee et al.^12^ used Random Forest (RF) modeling of neonatal vital statistics, in combination with sex, race, birth weight (BW) and GA. Their model outperformed the widely used Clinical Risk Index for Babies and the optimal logistic regression model. Addressing a different outcome, Lin et al. ^13^ applied ML to predict length of hospital stay among very low BW infants.

Although ML application to medical outcomes in preterm infants has become more prevalent, it still remains uncommon for neurodevelopmental prediction. A very ambitious neurodevelopmental study was executed by 165 ML model building “Fragile Families Challenge” teams in which many variables collected from birth to 9 were used to predict six life outcomes at age 15.^14^ Unfortunately, the highest R^2^ score attained was 0.23.

Better success has been achieved among preterm infants with less ambitious outcomes and using ML analyzed neuroimaging. Saha et al.^15^ analyzed MRIs from seventy-seven very preterm infants to forecast abnormal neuromotor development at 2 years. They dichotomized Neuro-Sensory Motor Developmental Assessment scores to implement a Convolutional Neural Network model achieving an F1-score of 68%. Vassar et al.^16^ applied ML to diffusion tensor imaging (DTI) fractional anisotropy, mean diffusivity, axial diffusivity, and radial diffusivity to predict language scores at 18–22 months, achieving 89% sensitivity/86% specificity. In a combinatorial approach, Valavani et al.^17^ predicted language delay in preterm infants at 2 years with neonatal clinical variables alone and with clinical and DTI data achieving balanced accuracies of 83% and 91%, respectively.

These studies indicate that applying ML to neonatal brain imaging can be effective in predicting neurodevelopmental outcomes. However, given the risks and costs of such neuroimaging, it is not yet universal practice even among very preterm infants. Predicting neurodevelopmental outcomes at 2 years, using only easily obtainable clinical variables, remains highly desirable. The goal of this study was to apply the RF algorithm to such variables to build an ML model to predict delay at 25 months of age in very preterm infants using Bayley Scales of Infant Development (BSID).^18,19^ Initially, we restricted modeling to perinatally available clinical variables; subsequently, we combined follow-up variables with these to determine the earliest age at which an infant’s 25-month developmental status can reliably be predicted.

## Methodology

### Participants

We leveraged 1109 longitudinal samples from a total of 3567 infants recruited from Richmond University Medical Center’s NICU.^20^ Selection criteria originally included any of these: BW < 1800 g; fetal distress with evidence of birth asphyxia; assisted ventilation (>48 h); persistent apnea/bradycardia; abnormal neurological signs; small for GA (<10th percentile BW for GA), intrauterine growth restriction (IUGR), or dysmature; multiple gestation (at least one meeting criteria or BW < 2000 g). Exclusion criteria were major congenital anomalies or chromosomal disorders. All infants with GA ≤ 33 weeks were selected. Years of births (YOBs) spanned 1989–2009. Participants comprised 47.2% females. They were 18.5% Latinx (black, white, Asian, and mixed Latinx) and 81.5% non-Latinx, with 25.3% black; 65.6% white; 5.2% Asian, 1.1% Indian, and 2.8% mixed. The research protocol was approved by IRBs of involved institutions. Signed informed consents were obtained from parents/guardians of all participants. Age was corrected for preterm birth.

### Measures/predictors

#### Perinatally available variables

There were many prenatal, perinatal, neonatal and maternal variables easily available to us, primarily from hospital medical records (see Supplementary Table 1 for complete list); some required interpretation. Intrauterine growth restriction (IUGR) was a standard score of BW for gestational age based upon norms (infants 22–50 weeks^21^). Cranial Ultrasound (CUS) scoring was based on interpretation of neonatal images. Abnormality/severity was classified as: (1) slight: prominent choroids; tiny choroid cysts; questionable abnormality; (2) mild: germinal matrix hemorrhage alone or with tiny cysts, intra-ventricular hemorrhage (IVH) alone (Papile Grade I); (3) moderate: IVH (Papile Grade II-III) alone or with cysts; ventriculomegaly ≤5 mm; (4) strong-severe: IVH grade III-IV; ventriculomegaly >5 mm; periventricular or parenchymal leukomalacia (PVL), hyperechoic echogenicity, or multiple cysts >3 mm; subarachnoid hemorrhage; cerebral edema >48 h with IVH or PVL; hydrocephalus >10 mm; hemorrhage or dilatation of III or IV ventricle; large or multiple porencephalic cysts, parenchymal hemorrhage or infarct; seizures requiring treatment.

#### Rapid neonatal neurobehavioral assessment

(RNNA)^22^ evaluates infants’ neurobehavioral function as newborns and 1-month-olds, and assesses visual and auditory attention, passive and elicited motor behaviors, state control, feeding, and jitteriness. It was developed for and normed on high-risk neonates and yields a general sum score based on degree and number of abnormalities Higher scores indicate more atypicalities.

#### BSIDs

BSIDs were completed at 4, 7, 10, 13, 16, 19, 22, and 25 months. Given that 25 months is the oldest age at which the BSID was administered, and our goal was to use ML to predict as far into the future as possible, similar to outcome ages in Valavani et al.^14^ and Saha et al.^12^ the 25-month assessment was our outcome variable. The BSID is an individually administered assessment measuring mental, psychomotor and behavioral development. Mental Developmental (MDI) and Psychomotor Developmental (PDI) Indices were analyzed. MDI reflects cognitive/language domains, while PDI captures fine/gross motor development.^23^ Both indices have a mean of 100 and standard deviation of 15. We dichotomized MDI and PDI scores, operationalizing moderate-to-severe impairment as a score <85. To understand the relationship between our MDI and PDI scores, we calculated correlations between them at each test age and found moderately positive correlations without dramatic differences across ages and with no discernible trend or linear relationship (see Supplementary Table 2).

### ML methodology

**Fig. 1:**
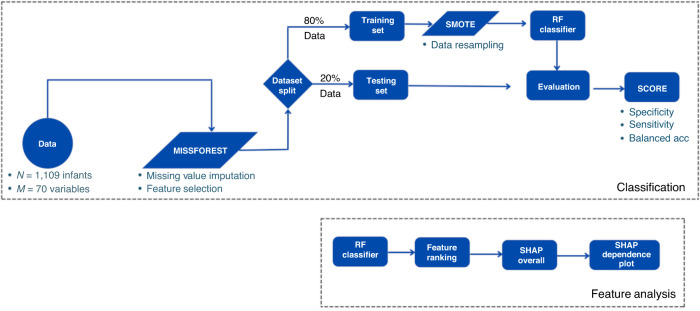
ML methodology flowchart. M is number of features (columns); N is sample size.

Our ML methodology (Fig. 1) was divided into classification and feature analysis. In classification, we first preprocessed our data to eliminate sparsity and to select important variables. Then RF was trained on the training set, which comprised 80% of the data. The other 20% was the test set on which we evaluated our model. In feature analysis, we used a trained model to extract feature ranking. Once the trained model was implemented, we used Shapley Additive exPlanations (SHAP)^24,25^ for interpretability. It showed feature effects on the predicted target value.

#### Data preprocessing

Three common challenges we faced in applying ML to our longitudinal data were sparse data, class imbalance and multicollinearity. Reasons for sparse data included losing families to follow up, skipped visits, untestable babies, and changes in data collection protocols. Most participants assessed at 25 months were not delayed, which resulted in class imbalance issues. Lastly, our data’s similar subscale tests with more than 80% correlated scores, created multicollinearity and high dimensionality by increasing feature numbers. To generalize our model and produce reliable results these challenges had to be addressed.

Given the prevalence of missing values in longitudinal studies, both training and test datasets should contain incomplete predictor variables for modeling real clinical settings. Our data sparsity/missing value ratios were: at-birth predictors (52 variables) 5.09%; RNNA predictors (2 variables) 15.06%; longitudinal predictors (14 Bayley scores) 57.63%. For each target MDI and PDI at month 25 the missing value percentage was 72%. To preserve information, we applied ML missing value imputation techniques to both the training set and test predictors, taking care to prevent data leakage. However, it was crucial to ensure the target values in the test set were solely comprised of genuine observations, without data augmentation or imputation. By preserving the test set’s authenticity, we sought to establish a fair and unbiased evaluation of our model’s performance against the benchmark. Due to a diversity of data types (continuous, multi-categorical, and binary), MissForest,^26^ a non-parametric missing value imputation technique based on RF, was chosen. MissForest^26^ initially inputs missing values with a statistical approach (mean/mode) and then refines them iteratively by prediction using an RF model. To mitigate data leakage in this process, we took measures to ensure the independence of our training and test data before implementing MissForest. Specifically, our training and test datasets were separated before applying the imputation technique. MissForest was then exclusively trained on the training set, ensuring that no information from the test set was utilized during the imputation process. Once imputation was complete, the technique was applied to the test data. These measures ensured our model’s performance was truly reflective of its generalization capabilities.

Imbalanced class data may cause low performance on minority class predictions and result in models that tend to overpredict majority class outcomes.^27^ Our target value class distribution (delayed vs non-delayed) was 14%/86%. This class imbalance was addressed by implementing an oversampling data balancing technique, Synthetic Minority Oversampling Technique (SMOTE),^28^ to create augmented data for the minority class. Figure 1 shows SMOTE^28^ applied only to the training set, ensuring our test data was original and that evaluation of the model’s accuracy would not be inflated by synthetic data.

**Fig. 2:**
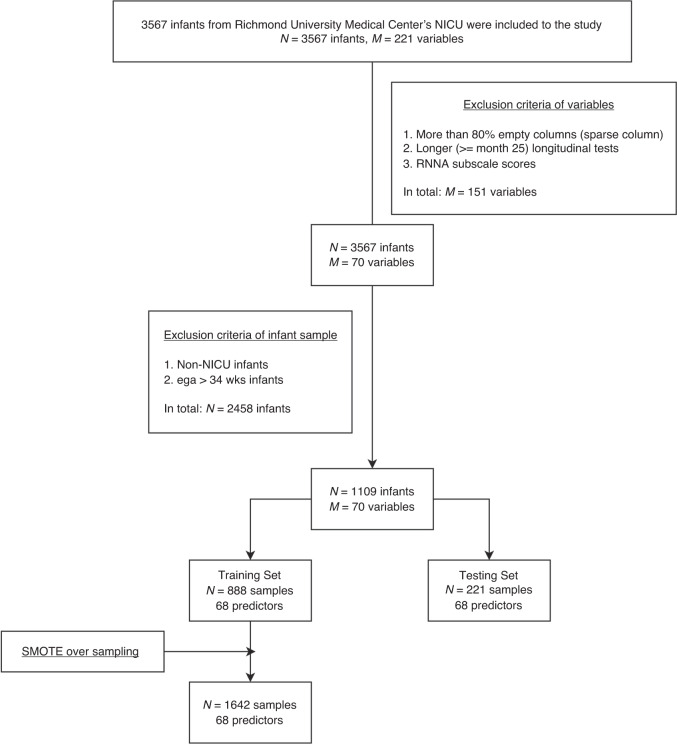
Data summary flowchart. M is number of features (columns); N is sample size.

Feature selection is a crucial step in achieving an optimal predictor set. To retain the most significant predictors while avoiding multicollinearity we first limited multicollinearity by setting a collinearity threshold of 0.8. Secondly, we utilized feature importance scores to identify the most relevant predictors. Subsequently, clinical personnel manually reviewed predictors, taking into consideration variable importance scores. Using these methods, we aimed to preserve the optimal predictor set. See Fig. 2 for preprocessing steps.

#### RF classifier

Seventy features and a binary category cut-off of 85 on MDI/PDI Scaled scores were examined with the RF Classifier model from Python’s sklearn package, (Fig. 1). Table 1 summarizes the design of the training and test datasets. RF is an ML algorithm utilizing an ensemble of decision trees to improve model generalizability and mitigate the risk of overfitting. Compared to base models like Logistic Regression, which assume linear relationships between variables and are sensitive to outliers, RF effectively handles high-dimensional, complex data sets and accommodates various data types. We compared RF to Logistic Regression, AdaBoost and XGBoost ML algorithms. RF outperformed them all (details in Supplementary Table 3). Given these factors, RF was chosen for our study.

**Table 1:** Train/Test data split with missing value and data sampling information.

	Training set (# samples: 888)		Test set (# samples: 221)	
	SMOTE	MissForest (Missing ratio)	SMOTE	MissForest (Missing ratio)
Target value	YES	YES (0.89)	NO	NO (0.00)
Predictors	YES	YES (0.21)	NO	YES (0.06)

Consistent with the goal of predicting, at birth using neonatal features, who will become delayed, an aim shared by many preterm studies,^15–17^ we began modeling with 52 perinatal variables. We then added longitudinal follow-up data to improve accuracy, resulting in 9 separate models with two target values each. Predictor information is summarized in Table 2 and was used twice, once for predicting delay from MDI and once from PDI scores. In addition to classification, we ran RF classifier to extract feature importance. While training the model using its random selection of features and multiple individual trees, RF assigns an importance score to each feature. Then feature ranking can be extracted from the trained model.

**Table 2:** Predictor information for models 1–9.

# Features	Variables	Model 1	Model 2	Model 3	Model 4	Model 5	Model 6	Model 7	Model 8	Model 9
52	At-birth	X	X	X	X	X	X	X	X	X
2	RNNA		X	X	X	X	X	X	X	X
2	Bayley 04			X						
2	Bayley 07				X					
2	Bayley 10					X				
2	Bayley 13						X			
2	Bayley 16							X		
2	Bayley 19								X	
2	Bayley 22									X
Total Variables		52	54	56	56	56	56	56	56	56

#### Evaluation metrics

Evaluation of model predictions utilized: sensitivity = True Positives/(True Positives + False Negatives), specificity = True Negatives/(True Negatives + False Positives), and balanced accuracy = (Sensitivity + Specificity)/2, which is considered a good measure of overall accuracy for imbalanced datasets like ours.^29^ In our study, true positives are correctly predicted delayed infants, sensitivity indicates the probability that a delayed infant will be predicted as delayed, and specificity is the probability that a non-delayed infant will be predicted as non-delayed.

#### SHAP feature analysis

We implemented SHAP explainability to help interpret how a given feature impacts our models’ predictions. ML explainability pertains to interpreting output from a complex non-linear model. SHAP methodology utilizes a game-theoretic framework to assign a “contribution” value to each feature in the model. This value signifies the degree to which a given feature influences the model’s output, allowing SHAP to explain the correlation between a particular feature and the prediction. Thus, the output obtained using SHAP may be referred to as an explainable ML result. It not only addresses feature importance, but the direction of its effect. SHAP also provides an explanation based on the magnitude of feature attributions. Feature effects on predictions may be linear or non-linear. SHAP plots depict these relationships, accordingly, allowing a more comprehensive understanding of the influence of each feature. SHAP represents its output decisions visually using figures.

## Results

### Model 1–prediction using only perinatally available variables

Table 3 reports the top 15 features for Model 1; Table 4 reports means for those features the top 15 predictors were very similar for both mental and psychomotor developmental models.

**Table 3:** Top 15 most important features from model 1.

	Mental Delay (MDI)	Psychomotor Delay (PDI)
1	Birth year	Birth year
2	Mom education	Days in hospital
3	Days in hospital	Mom education
4	MgSO4 (Yes/No)	Abnormal CUS
5	Days intubated	Days intubated
6	Birth weight	Gestational age (wks)
7	Abnormal CUS	Mom age (yrs)
8	Gestational age (wks)	Birth weight
9	Mom age (yrs)	MgSO4 (Yes/No)
10	Intrauterine growth restriction	1 min Apgar score
11	Toxicology done	Late/No prenatal care (Yes/No)
12	Late/No prenatal care (Yes/No)	Intrauterine growth restriction
13	Head circumference	Patent ductus arteriosus
14	Days on CPAP	Head circumference
15	1 min Apgar score	Days on CPAP

**Table 4:** Participant characteristics for model 1 top 15 features and 2 outcomes.

	Total (*N* = 1109)	Mental Delay (MDI < 85)	NO Mental Delay	Psychomotor Delay (PDI < 85)	NO Psychomotor Delay
Birth Yr < 2000 Birth Yr ≥ 2000	37.1% 62.9%	41.4% 36.5%	58.6% 63.5%	58.6% 39.2%	41.4% 60.8%
Mom’s education	14.3 ± 2.4 (10–22)	13.3 ± 2.6 (6–21)	14.3 ± 2.4 (10–22)	13.8 ± 2.7 (6–22)	14.1 ± 2.4 (10–19)
Days in hospital	43.3 ± 30.5 (5–247)	55.6 ± 35.2 (5–180)	40.9 ± 25.2 (5–143)	56.6 ± 33.1 (6–180)	36.8 ± 23.6 (5-106)
YES MgSO_4_	35.5%	23.2%	37.6%	23.5%	40.1%
NO MgSO_4_	25.9%	23.1%	16.9%	19.0%	19.8%
Missing	38.6%	53.7%	45.5%	57.5%	40.1%
Days intubated	6.6 ± 13.0 (0–90)	10.4 ± 15.8 (0–90)	5.5 ± 9.8 (0–45)	10.3 ± 15.1 (0–90)	4.5 ± 8.9 (0–44)
Birth weight (gm)	1398 ± 437 (369–2948)	1283 ± 433 (539–2268)	1441 ± 424 (510–2608)	1278 ± 432 (539–2353)	1478 ± 414 (510–2608)
Abnormal CUS					
None	54.6%	45.4%	57.1%	41.2%	63.7%
Slight	24.3%	18.2%	21.2%	18.3%	21.7%
Mild	10.9%	17.4%	11.1%	19.6%	7.6%
Moderate	5.1%	8.3%	6.4%	9.8%	4.5%
Severe	5.1%	10.7%	4.2%	11.1%	2.5%
Gestational age (wks)	30.3 ± 2.5 (23–33)	29.6 ± 2.7 (24–33)	30.4 ± 2.2 (24–33)	29.5 ± 2.6 (24–33)	30.7 ± 2.1 (24–33)
Mom’s age (yrs)	30.0 ± 6.0 (15.0–49.9)	30.1 ± 6.1 (15.6–45.5)	31.1 ± 6.0 (16.6–49.9)	30.9 ± 5.8 (17.0–49.9)	30.5 ± 6.2 (15.6–44.2)
IUGR measure	−0.70 ± +1.1 (−4.7 - +4.9)	−0.79 ± +1.0 (−3.1 - +2.3)	−0.62 ± +1.0 (−4.7 - +2.0)	−0.71 ± +1.1 (−3.1 - +2.3)	−0.66 ± +1.0 (−4.7 - +2.0)
Toxicology					
Done	17.2%	15.7%	19.0%	15.0%	20.4%
Not Done	66.8%	50.4%	59.3%	51.0%	60.5%
Missing	16.0%	33.9%	21.7%	34.0%	19.1%
Prenatal Care					
Yes	87.1%	77.0%	90.4%	85.2%	85.3%
No/late	12.9%	23.0%	9.6%	14.8%	14.7%
HC (cm)	28.1 ± 2.7 (21–35)	27.5 ± 3.0 (21–36)	28.1 ± 2.7 (21–35)	27.3 ± 3.0 (21–36)	28.4 ± 2.6 (21–35)
Days on CPAP	11.2 ± 14.3 (0–135)	11.9 ± 13.6 (0–72)	9.2 ± 12.0 (0–67)	12.1 ± 13.6 (0–72)	8.4 ± 11.5 (0–60)
Apgar 1 min	6.5 ± 1.9 (0–9)	6.0 ± 2.2 (0–9)	6.6 ± 2.0 (1–9)	5.8 ± 2.2 (0–9)	6.9 ± 1.7 (1–9)
YES PDA NO PDA	34.6% 65.4%	45.5% 54.5%	38.7% 61.3%	55.8% 44.2%	28.7% 71.3%
Mental Dev Index (MDI)	N/A	72.4 ± 11.2 (49–84)	101.8 ± 10.5 (86–137)	81.7 ± 17.6 (66–137)	98.8 ± 13.7 (66–137)
Psychomotor Dev Index (PDI)	N/A	75.4 ± 16.0 (49–110)	90.7 ± 12.9 (57–133)	71.4 ± 10.1 (49–84)	97.7 ± 7.9 (85–133)

The classification model accuracy results for Model 1 cognitive/verbal and fine/gross motor delay respectively were sensitivity = 91.9%; 93.4%, specificity = 31.8%; 29.4%, and balanced accuracy = 61.9%; 61.4%. The high sensitivities indicate that we miss few infants who go on to be delayed, but low specificities indicate there are many false alarms—we predict infants will be delayed who will not be.

### Model 2–9 prediction using perinatally available and longitudinal assessment variables

Results from adding longitudinal data to the modeling process are summarized in Table 5 for both MDI and PDI targets. We expected that including assessments administered closer to the 25-month targets would progressively improve the models’ balanced accuracies. However, there was no linear relationship across balanced accuracies indicating this. Model 8, which included month 19 MDI and PDI scores, had the highest balanced accuracy.

**Table 5:** Model accuracies for target value of mental and psychomotor delay at month 25.

	Mental Development (MDI)			Psychomotor Development (PDI)		
Model/Age	Sensitivity	Specificity	Balanced Accuracy	Sensitivity	Specificity	Balanced Accuracy
1 (birth)	91.9	31.8	61.9	93.4	29.4	61.4
2 (1 mo)	86.8	31.3	59.0	95.3	28.3	61.8
3 (4 mo)	86.0	35.5	60.7	**96.0**	30.2	63.1
4 (7 mo)	92.3	35.6	63.9	95.3	30.4	62.9
5 (10 mo)	91.7	33.9	62.8	94.7	30.4	62.6
6 (13 mo)	**94.4**	34.5	64.5	93.3	36.0	64.7
7 (16 mo)	89.6	35.8	62.7	95.5	40.2	67.9
8 (19 mo)	88.7	**54.8**	**71.7**	93.9	**51.7**	**72.8**
9 (22 mo)	93.8	39.8	66.8	87.1	43.9	65.5

Emphasized in bold are the highest values.

### SHAP analyses and model explainability

We applied SHAP explainability to Model 1, which used clinical perinatally available variables as predictors, and Model 8, the model with the highest balanced accuracy. We re-trained these models’ predictors to apply SHAP analysis. In SHAP’s pictorial results red represents higher values of a feature while blue represents lower values. Mixed or speckled colors reflect a non-linear relationship between the variable and prediction of delay. In our analyses, a target value of 0 was designated as non-delayed and 1 as delayed. Thus, a positive SHAP value (*x*-axis) indicates a greater likelihood of predicting delay.

**Fig. 3:**
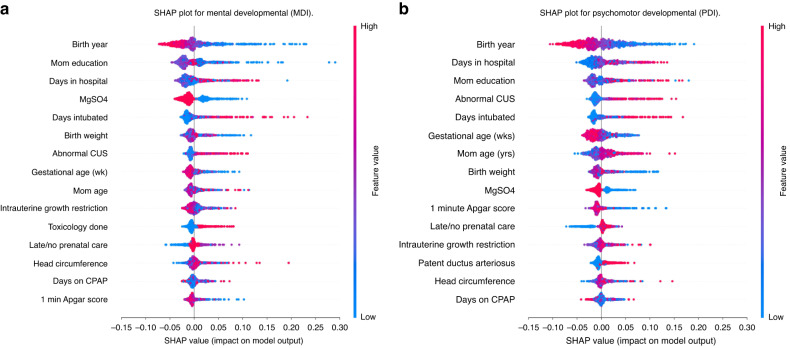
SHAP summary plot for model 1. Panel **a** corresponds to the Mental Developmental (MDI), while panel **b**) represents the SHAP plot for the Psychomotor Developmental (PDI). A positive SHAP value (*x*-axis) indicates a higher likelihood of a delay. MgSO4 (0 = no, 1 = yes); CUS (higher values = greater abnormality); IUGR (lower values = less growth restricted); Toxicology done (0 = no, 1 = yes); Late/no prenatal care (0 = yes, 1 = no or late); PDA (0 = no, 1 = yes).

**Fig. 4:**
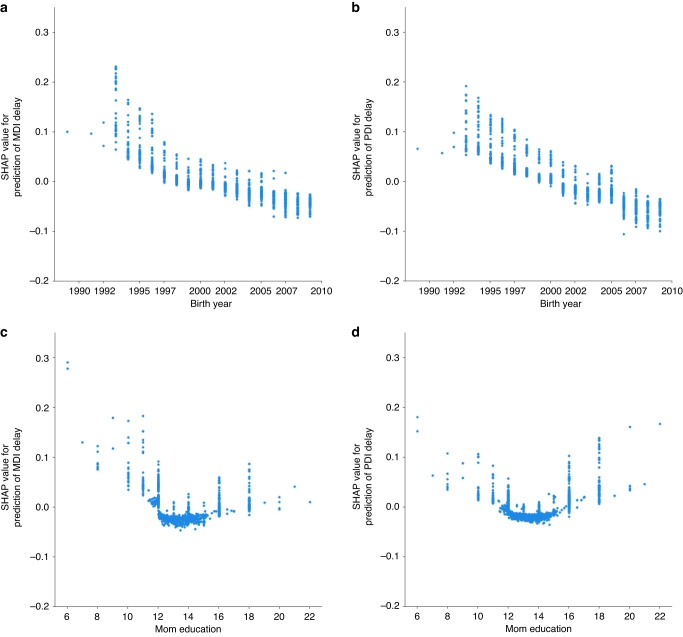
Dependence plot for individual at-birth features. A higher SHAP value (*y*-axis) represents higher likelihood of predicting delay (**a**, **b**) effect of YOB on Mental Development Index (MDI) and Psychomotor Development Index (PDI) delay, respectively. **c**, **d** effect of mom’s education on MDI and PDI delay, respectively.

**Fig. 5:**
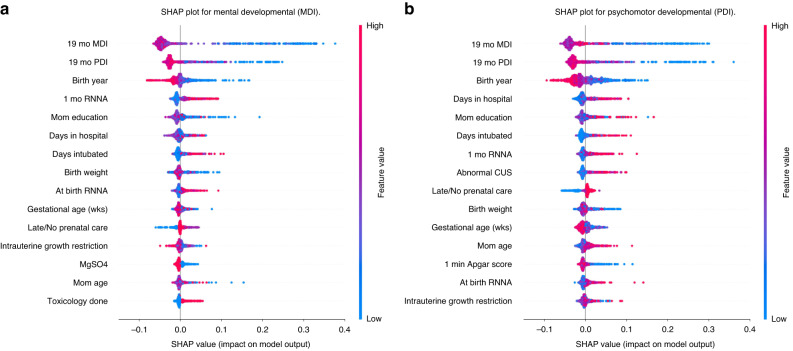
SHAP summary plot for model 8. Panel **a**) corresponds to the Mental Developmental (MDI), while panel **b**) represents the SHAP plot for the Psychomotor Developmental (PDI). A positive SHAP value (x-axis) indicates a higher likelihood of delay. RNNA (higher values = more abnormalities); CUS (higher values = more abnormality); Late/no prenatal care (0 = yes, 1 = no or late); IUGR (lower values = less growth restricted); MgSO4 (0 = no, 1 = yes); Toxicology done (0 = no, 1 = yes); PDA (0 = no, 1 = yes).

Figures 3 and 5 depict SHAP values for Models 1 and 8, respectively, representing the top 15 features and the direction and magnitude of their effects on the targets for mental and psychomotor development. In Fig. 3, a lower value of YOB (blue) contributed to a prediction of both mental and psychomotor developmental delay, and a higher value (red) contributed to not being delayed. Expressed differently, being born earlier contributed more to a prediction of being delayed while being born more recently contributed to a prediction of non-delayed. Similarly, in Fig. 5, a lower value (blue) of 19-mo MDI contributed to a prediction of delay for both MDI and PDI. One last example, in Figs. 3 and 5, higher values (red) of days intubated contributed to the likelihood of predicting delay. In both models 1 and 8 SHAP summary plots, each feature produces similar output. That was expected because SHAP calculates the marginal effect of each predictor individually Fig. 4.

**Fig. 6:**
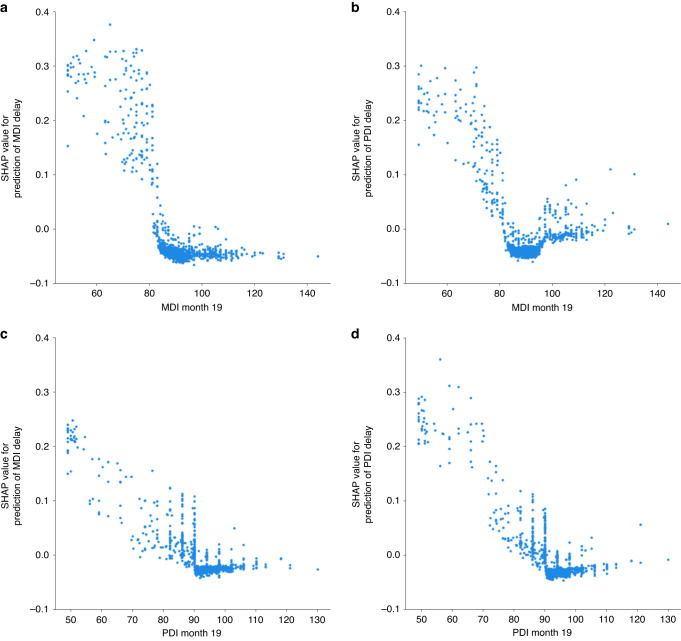
Dependence plot for individual temporal features. A higher SHAP value (*y*-axis) represents the higher likelihood of delay (**a**, **b**) effect of Bayley Mental Development Index (MDI) at month 19 on (MDI and Psychomotor Development Index (PDI) delay respectively. **c**, **d** effect of PDI at month 19 on MDI and PDI delay, respectively.

SHAP also can produce dependence plots which provide detailed depictions of individual features’ marginal effects. Dependence plots for YOB and mom’s education are in Fig. 5. For YOB, there is a clear linear decrease in SHAP values as YOB becomes more recent. The effect of mom’s education is more complex, having almost no effect on cognitive/verbal delay except when education is less than high school, but for fine/gross motor delay, the effect is u-shaped. Both lower and higher education are associated with delay. Dependence plots for 19-month MDI and PDI (Fig. 6), show that delayed 19-month scores (≤85) contributed to delay at 25 months in an almost linear fashion, but scores above 85 contribute little to a change in SHAP values. (See Supplementary Figs. 1–13 for dependence plots for other top predictors).

## Discussion

Providing intervention services to very preterm infants at the earliest possible time is important to maximizing their potential.^30–33^ Using a diverse, retrospective dataset of infants (*N* = 1109) born over a 20-year period, we applied ML to predict neurodevelopmental delay at 25 months of age. We succeeded in developing a model that predicted likely cognitive/verbal and fine/gross motor delay ½ year in advance, potentially allowing intervention to start that much sooner. For 25-month-olds, that represents one quarter of their lives.

It would be ideal to predict who would be delayed as early as the newborn period. Considering that goal, we began ML modeling using newborn data. Of 52 perinatal variables, the top predictors included: YOB, mom’s education, days in hospital, antenatal MgSO_4,_ days intubated, BW, abnormal CUS, GA, mom’s age, IUGR, toxicology done, no/late prenatal care, head circumference, 1 min Apgar score, and PDA. There was extensive feature overlap for prediction of cognitive/verbal and fine/gross motor delay. Balanced accuracies for these models fell short of clinical application. High sensitivity was offset by low specificity. They were good at not missing infants who would be delayed but fell short on predicting those who would not be delayed.

Similar to Ambalavanan’s^34^ and Salganik’s.^14^ approach, but at different time scales, we then added longitudinal features from follow-up assessments to improve accuracy, resulting in 9 models with two target values each. In contrast to Salganik et al.^14^ who were predicting outcome at older ages, this was somewhat successful. Contrary to expectations, there was no linear increase in the models’ accuracies as the assessments included were nearer the 25-month target. Highest balanced accuracies (72%/73%) were achieved with 19 not 22-month scores. SHAP dependence plots provided a detailed picture of 19-month score effects. Scores above ~85 contributed little to predicting 25-month delay, but lower, delayed scores increased the likelihood of delay. This was most prominent for MDI scores where the increase in predicting delay dropped almost linearly for scores below 85, but was flat for those above, suggesting if a child is delayed at 19-months, they will likely continue to be delayed at 25 months. If intervention has not already begun, it should be started immediately rather than waiting for the child to “grow out of it” or “catch up” as is sometimes recommended. Although it is beyond the scope of our study to answer the question of why the 19-month assessment seemed more predictive than at other ages, we suggest it may be related to cascading effects on subsequent development of a delay in the 18–19 month “vocabulary growth spurt”.^35^ Related to this and to the timeline used, we conducted supplementary analyses investigating effects of altering the target value timeline, such as changing it from month 4 to month 25. A series of changes in the target values did not exhibit a linear relationship with accuracy performance, but feature importance rankings derived from clinical at-birth variables remained notably consistent regardless of the target value. See Supplementary Table 14 for details.

Our study was ambitious in attempting to apply ML to a diverse sample collected over 20 years with the potential advantage of producing more transportable models, but possibly exacerbating other challenges often accompanying longitudinal data. To train the ML model, we implemented data augmentation which addressed the imbalance between delayed and non-delayed children. Care was given to assure our test dataset consisted only of original values in the target field for both data imputation and SMOTE. Thus, although we synthesized data to impute missing entries and address class imbalance, evaluation was only performed by comparing the predicted target values with actual target values, not synthetic ones. This may help explain our less favorable results compared to work such as Valavani, et al.^17^ which predicts preterm verbal delay with balanced accuracies of 83% (clinical variables) and 91% (clinical and DTI variables). Their validation assessment adopted SMOTE to resample their entire dataset, synthetic target values as well as real ones. When we implemented their validation scheme, our balanced accuracies increased by over 20%, to 86%/86% (MDI/PDI delay - perinatal features); and to 92%/88% (MDI/PDI - perinatal features and 19-month scores). However, given that synthetic data is not guaranteed to reflect the true population distribution, using it to evaluate accuracy can be misleading. Adopting a more rigorous approach to model assessment increases the possibility that the model will be transportable.

The focus of our study was early prediction of neurodevelopmental delay. In ML it is the model in its entirety—the black box – that accomplishes this. However, to clinicians and developmentalists, feature ranking and SHAP’s further description of the magnitude and direction of feature effects are of interest. That said, many key features emerging from our models are consistent with recent ML preterm research including antenatal MgSO_4,_ days intubated, BW, GA, and maternal age.^17^ CUS, a high-ranking feature in our models, is quite different from AI analyzed MRI/DTI images, important in other preterm outcome modeling.^15–17^ However, SHAP’s association of CUS damage with greater likelihood of delay is consistent with this research and with the role of CNS injury frequently reported in non-ML studies.^36,37^ Our study was limited by reliance on clinical rather than AI interpretations of our CUSs. Direct AI analyses of CUSs, which are universally performed in very preterm infants, is a goal for future work.

Along with sample breadth, another distinctive aspect of our study was modeling multiple outcome domains in the same data. The few ML studies closely related to ours have focused on a single domain or on combined outcomes. Valavani et al.^17^ and Vassar et al.^16^ predicted verbal delay, while Saha et al.^15^ restricted predictions to the motor domain (Neuro-sensory Motor Developmental Assessment), and Ambalavanan et al.^34^ combined MDI, death, cerebral palsy, deafness, or blindness into one outcome. We applied the same set of models to both cognitive/verbal delay and fine/gross motor delay. This potentially allowed us to examine how perinatal and longitudinal features differentially affected these separate outcomes. What is clear from our results is that the top predictive features for both are very closely aligned and exhibit similar explainability in SHAP analyses. Although specialized interventions are needed to address delays in these different domains, it may be possible to broadly target infants most likely to run into difficulty with a similar set of red flags.

In the context of an epigenetic developmental perspective, successful prediction of outcomes past the perinatal stage will likely have limitations without incorporating environmental and longitudinal variables, except in cases of extreme brain injury. And the further into the future the prediction, the greater the challenge. Most of our top features were clinical, with only a limited few - maternal age, maternal education (considered indicative of perinatal SES,^38^ toxicology report requested, and no/poor prenatal care - likely to reflect environmental-biological interactions. Consistent with our results, maternal education has often been associated with neurodevelopmental outcomes.^39,40^ However our SHAP plots suggested, especially for cognitive/verbal delay, the effect was not linear but strongest if mom’s education was high school or less. A feature less commonly considered in similar studies, perhaps due to apriori clinical rather than ML selection, is no/poor prenatal care. Prenatal care is most often researched in relation to low BW and other adverse obstetric, not neurodevelopmental, outcomes. Surprisingly, even from the obstetric viewpoint, the importance of prenatal care has been debated.^41–43^ Our results clearly connect late/no prenatal care with increased likelihood of predicting cognitive/verbal and fine/gross motor delay, thus supporting the importance of prenatal care to positive neurodevelopmental outcomes, although we recognize that this variable can be a surrogate for a spectrum of additional environmental variables, particularly in a society where access is unequal.

YOB ranked first as a predictor in the at-birth only model and third in the 19-month model. The high ranking of YOB is likely related to the 20-year time span encompassed by our data. Year of birth is not typically included in risk modeling. Its appearance as a top feature suggests practice guidelines for ML modeling. Models used clinically will require periodic updating as medical/clinical practices and technology advance. Given the importance of YOB to our study, we quantitatively assessed its impact on model performance. We re-ran our classification model omitting YOB. This reduced RF balanced accuracy approximately 5% for mental and motor delay. SHAP visualizations of YOB depict a clear linear decline in likelihood of delay as YOB moves from the 1990s into the 21st century, with more recent YOB less likely to predict delay. It suggests that infants in our study born more recently did better in both cognitive/verbal and fine/gross motor domains. This presents a positive picture for the field of pediatrics, particularly neonatology, suggesting that continued improvements in medical care for infants born very preterm have resulted in better neurodevelopmental outcomes. Although our modeling strongly suggests this our study is limited in that the data are from one medical center and we cannot conclusively rule out other explanations. Nonetheless, this is a positive take home message consistent with numerous other reports.^44–46^

## Conclusion

To provide the earliest intervention to very preterm infants, it would be ideal to predict, at birth, who will be delayed and in need of services. The combination of both longitudinal and perinatal features produced an ML model that predicted 25-month delay at 19 months, potentially rolling back intervention by ½ year, 25% of the child’s life. Despite ML modeling using 52 perinatal variables, the goal of at-birth prediction remained elusive. Our models lacked clinical-ready precision, with high sensitivity but low specificity, missing few who would be delayed but overpredicting delay. From a developmental epigenetic viewpoint, it is not surprising that perinatally available variables have limitations in accurately predicting subsequent development. The precision of future ML models might be improved by applying AI interpretation to perinatal CUSs and by including additional longitudinal variables that capture ongoing environmental interactions.

### Supplementary Information

Supplementary Material

## Data Availability

The data that support the findings of this study are available from The Research Foundation for Mental Hygiene, but restrictions apply to the availability of these data, which were used under license for the current study, and so are not publicly available. Data are, however, available from the authors upon reasonable request and with permission of The Research Foundation for Mental Hygiene.
