# Supplementary material for: Predicting mental and psychomotor delay in very pre-term infants using machine learning
Source: Pediatr Res. 2023 Jul 27;95(3):668–78. doi: 10.1038/s41390-023-02713-z (PMC10899098; doi:10.1038/s41390-023-02713-z)
Supplement: Supplementary file 1 — Supplementary Material [file 41390_2023_2713_MOESM1_ESM.pdf]

Supplementary Material

Predicting Mental and Psychomotor Delay in Very Pre-term Infants using Machine Learning

Gözde M. Demirci, Phyllis M. Kittler, Ha T.T. Phan, Anne D. Gordon, Michael J. Flory, Santosh M. Parab, & Chia-Ling Tsai

Supplementary methods

Additional SHAP Visualizations

We provide additional information and visualizations to support the findings presented in the main manuscript. Specifically, we present dependence plots for the remaining 13 of the 15 most important features identified by SHAP (SHapley Additive exPlanations) analysis qq. v., Supplementary Figure 1 - 13. These plots show the relationship between the feature values and the target values of mental development index (MDI) and psychomotor development index (PDI) for each feature. These results provide further insight into the factors that contribute to the prediction of MDI and PDI values as identified by our machine learning model. In the figure interpretation, the likelihood of predicting an infant as delayed increases as the SHAP value (y-axis) increases.

Justification of Target Variable Timeline

To assess the impact of changes to the timeline on target values, we conducted a supplementary series of analyses. Specifically, we investigated the effects of altering the timeline on the target value, for example, from month 25 to month 22. Our findings reveal that changes in the duration of the target value do not exhibit a linear relationship with the accuracy performance. However, we observed that the feature importance rankings derived from clinical at-birth variables were notably consistent, indicating that the same set of features have a consistent impact on the target values. Therefore, we sought to extend our analysis further by exploring the changes in the

characteristics of the important features. To achieve this, we leveraged the SHAP results for additional insight. The details of the supplementary experiment can be seen in Supplementary Figure 14.

Supplementary Tables

Supplementary Table 1. Detailed predictor information used in ML models.

At Birth			RNNA	Mental Dev Index (MDI)	Psychomotor Dev Index (PDI)
abnormal placenta	barbiturate exposure	1 minute Apgar score	newborn score	month 4	month 4
apnea	benzodiazepine exposure	5 minute Apgar score	month 1 score	month 7	month 7
asphyxia/hypoxia	cannabis exposure	head circumference (cm)		month 10	month 10
autism	crack/cocaine exposure	birth weight (gm)		month 13	month 13
autism sib	opioid exposure	gestational age (wks)		month 16	month 16
maternal temperature	methadone exposure	intrauterine growth restriction (IUGR) measure		month 19	month 19
bradycardia	toxicology report requested	days intubated		month 22	month 22
breech presentation	nicotine exposure	days on CPAP			
c-section	beer/wine exposure	days in hospital			
emergency C-section	necrotizing enterocolitis	birth year			
Abnormal CUS	metabolic acidosis	hyperbilirubinemia			
decelerations	late/no prenatal care	antenatal $MgSO_4$			
discordant twin	sex	mom age (yrs)			
HIV positive	meconium aspiration	mom education			
diabetic mom	patent ductus arteriosis	# of infants in birth			
administered phenobarbitol	respiratory distress	birth order for multiple birth			
seizures	hyaline membrane disease				
steroids	pre-eclampsia				

Supplementary Table 2. Correlation between MDI and PDI at each period. Table displays three population groups from the study: the total sample, those with mental development delays at month 25, and those with psychomotor development delays at month 25. The "Mental" and "Psychomotor" columns displaying the average and standard deviation of developmental scores for mental and psychomotor domains at specific age intervals.

MONTH	MENTAL	PSYCHOMOTOR	CORRELATION
**TOTAL SAMPLE**			
4	101.0 (±11.5)	95.5 (±10.2)	0.67
7	99.3 (±9.1)	93.5 (±14.0)	0.41
10	99.6 (±9.7)	96.5 (±14.9)	0.4
13	101.4 (±13.4)	92.3 (±19.6)	0.53
16	95.6 (±14.6)	94.9 (±18.1)	0.58
19	89.8 (±16.6)	89.8 (±14.2)	0.53
22	89.2 (±18.2)	87.9 (±14.3)	0.5
25	90.4 (±17.9)	84.7 (±16.0)	0.55
**INFANTS DELAYED ON MENTAL AT 25 MO**			
4	98.7 (±11.7)	93.2 (±11.0)	0.64
7	96.9 (±10.5)	89.6 (±15.9)	0.44
10	96.7 (±9.8)	93.8 (±17.3)	0.38
13	96.2 (±12.2)	85.3 (±19.8)	0.6
16	87.7 (±14.9)	87.3 (±21.4)	0.65
19	78.6 (±13.5)	83.6 (±15.8)	0.56
22	75.8 (±12.8)	82.1 (±15.5)	0.45
25	72.4 (±11.2)	75.4 (±16.0)	0.49
**INFANTS DELAYED ON PSYCHOMOTOR AT 25 MO**			
4	98.8 (±12.0)	93.3 (±10.7)	0.66
7	97.5 (±9.5)	89.9 (±15.6)	0.39
10	98.1 (±9.9)	93.2 (±16.3)	0.49
13	98.2 (±12.1)	83.4 (±17.8)	0.4
16	90.2 (±14.1)	86.9 (±19.9)	0.57
19	84.7 (±16.4)	83.8 (±15.8)	0.49
22	82.9 (±17.0)	80.9 (±12.4)	0.43
25	81.7 (±17.6)	71.4 (±10.1)	0.49

Supplementary Table 3. ML algorithms comparison (% balanced accuracy). To ensure objectivity, the model hyperparameters and data settings for all the algorithms were standardized. The clinical at-birth variables were used as predictors, while the target values were mental and psychomotor developmental delay at month 25 respectively.

Model/Age	Mental Development	Psychomotor Development
**Logistic Regression**	55.14 %	58.12 %
**AdaBoost**	55.67 %	58.25 %
**XGBoost**	54.70 %	56.30 %
**Random Forest**	61.9 %	61.4 %

Supplementary Figures

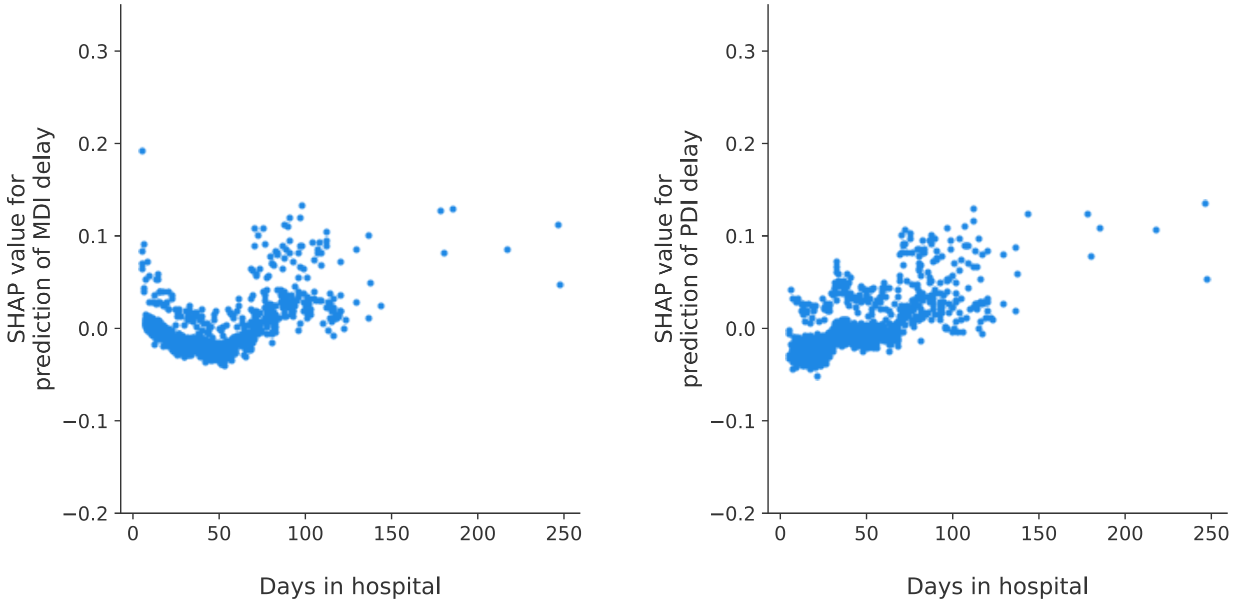

Supplementary Figure 1. The effect of days in hospital on mental developmental (MDI) and psychomotor developmental (PDI) delay respectively.

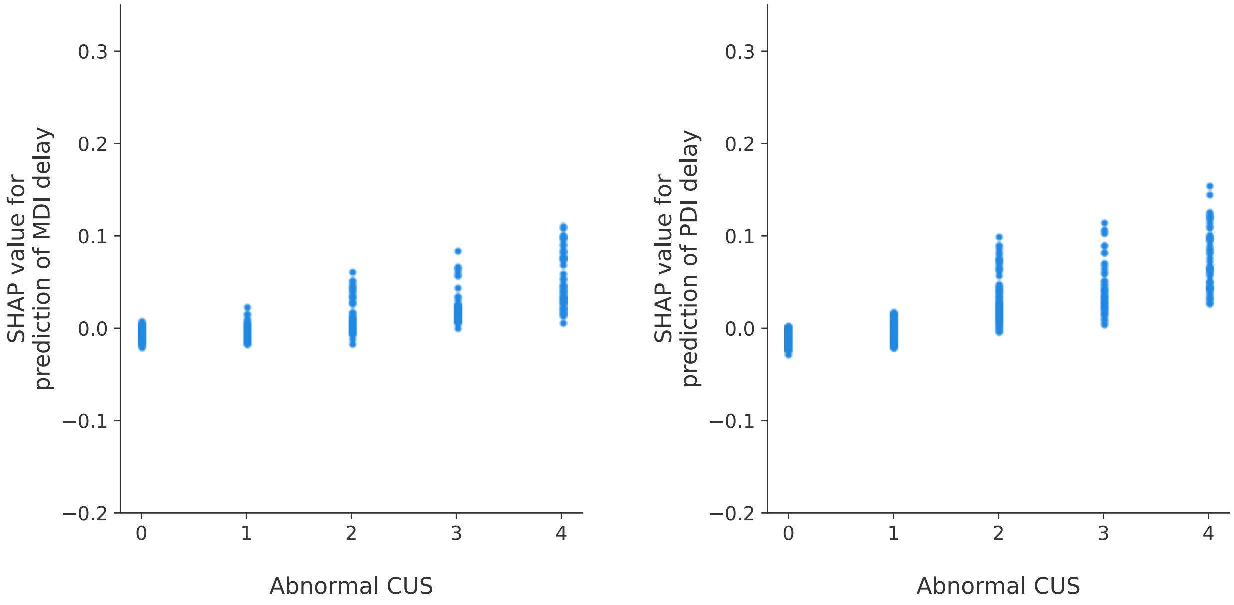

Supplementary Figure 2. The effect of abnormal CUS on mental developmental (MDI) and psychomotor developmental (PDI) delay respectively. Higher values indicate more severe abnormality.

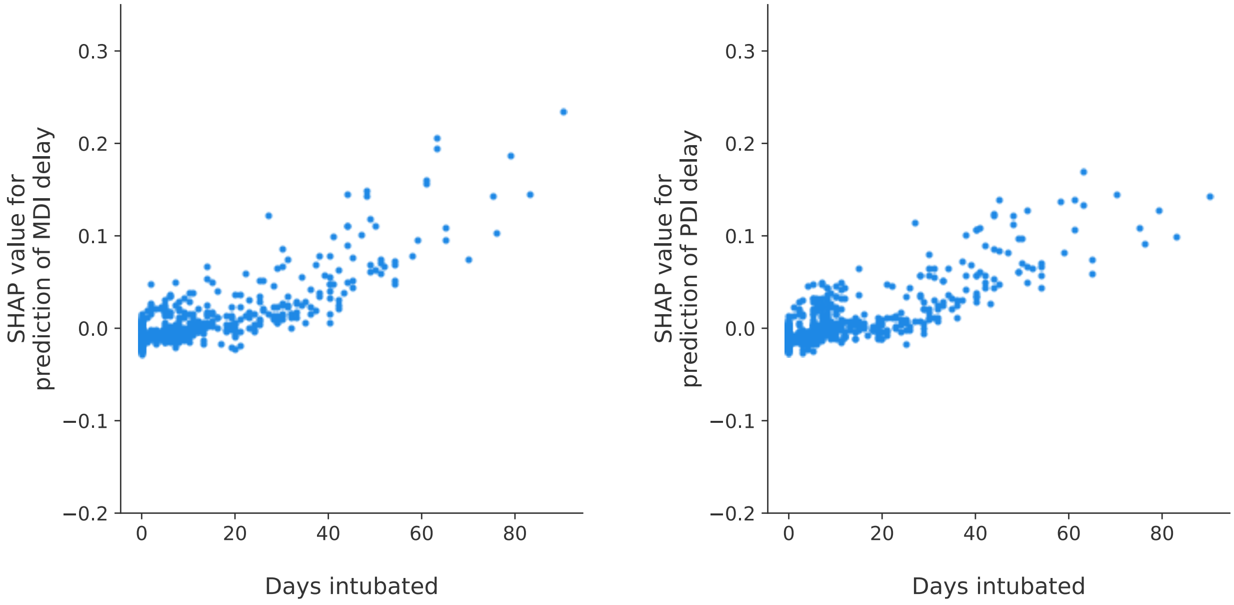

Supplementary Figure 3. The effect of days intubated on mental developmental (MDI) and psychomotor developmental (PDI) delay respectively.

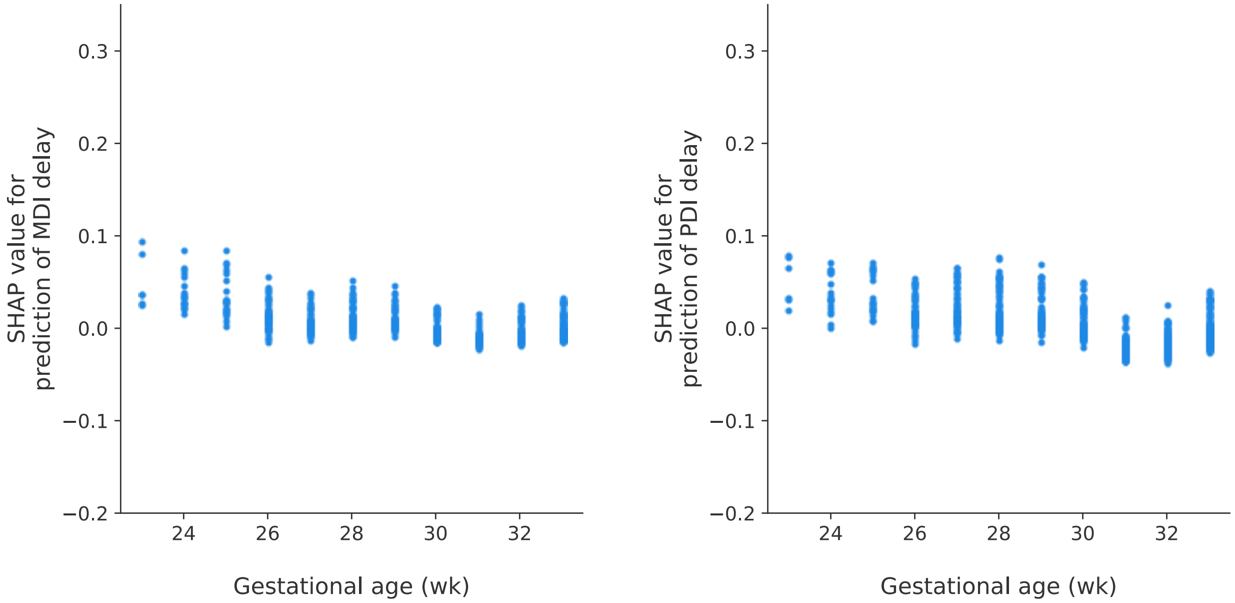

Supplementary Figure 4. The effect of gestational age on mental developmental (MDI) and psychomotor developmental (PDI) delay respectively.

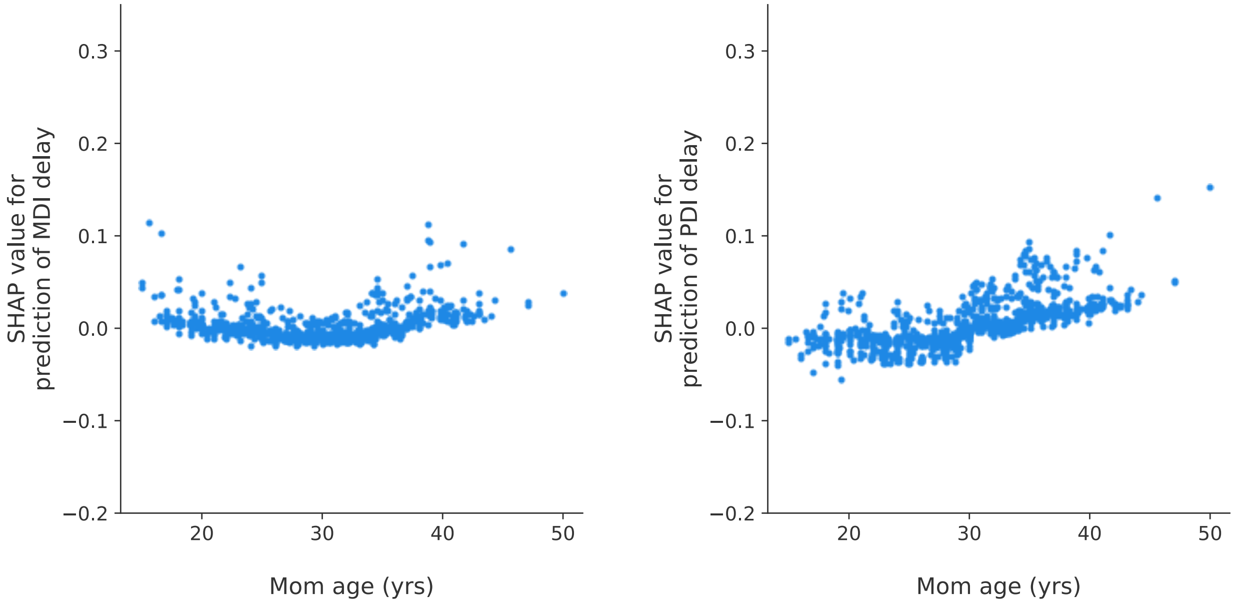

Supplementary Figure 5. The effect of mother age on mental developmental (MDI) and psychomotor developmental (PDI) delay respectively.

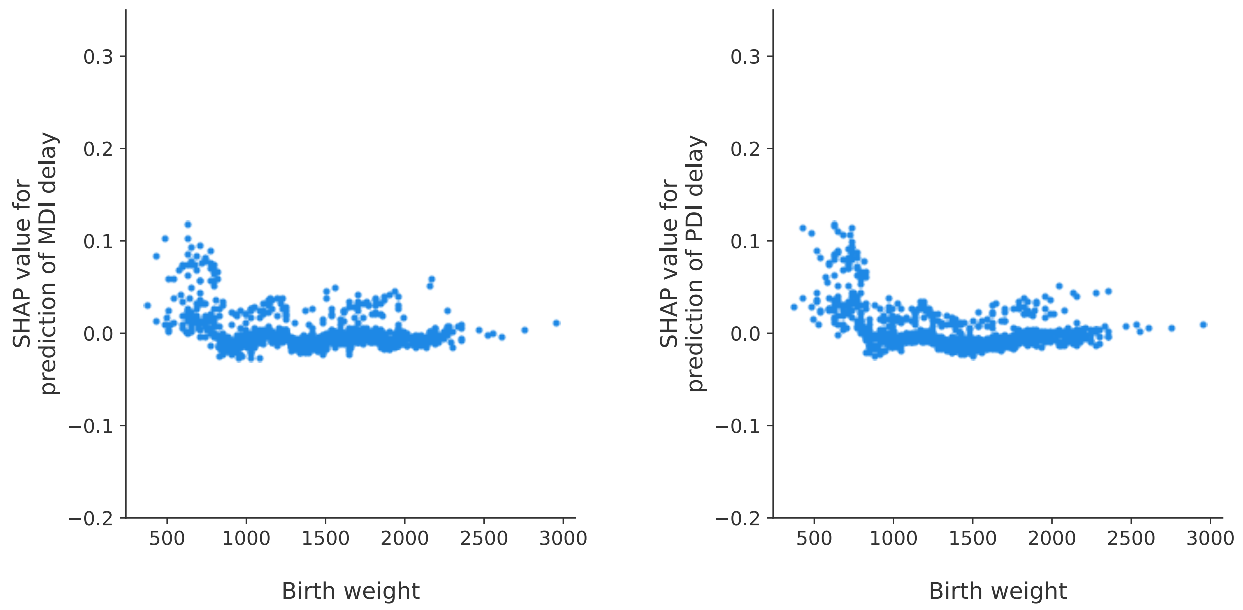

Supplementary Figure 6. The effect of birth weight on mental developmental (MDI) and psychomotor developmental (PDI) delay respectively.

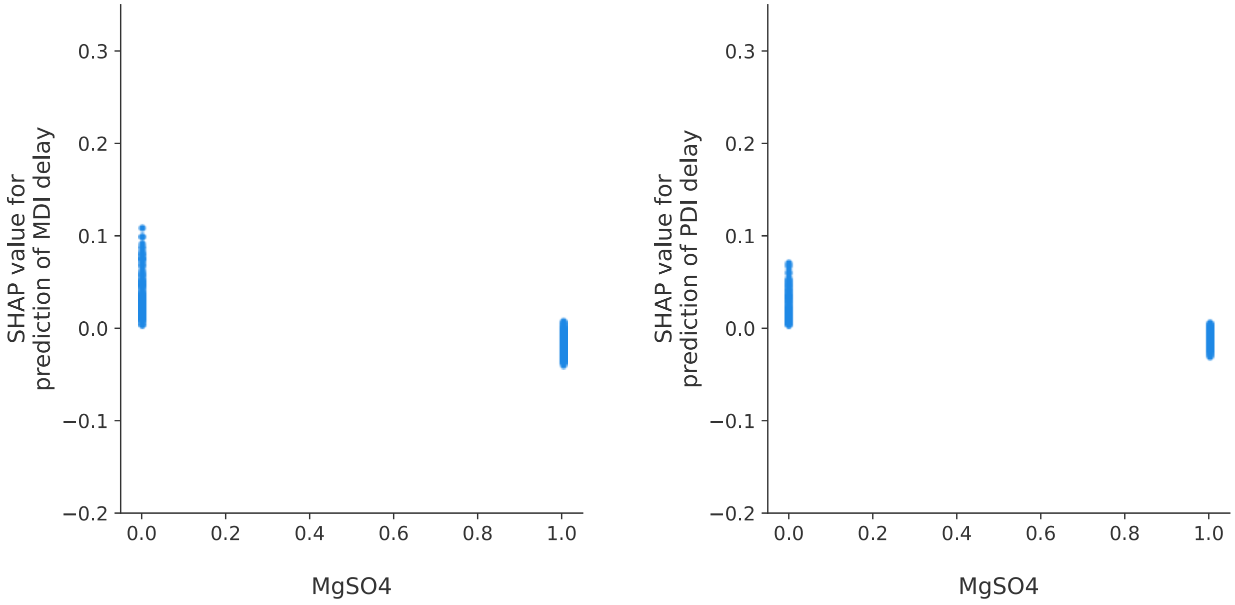

Supplementary Figure 7. The effect of $MgSO_4$ on mental developmental (MDI) and psychomotor developmental (PDI) delay respectively. 0 is no $MgSO_4$; 1 is treated with $MgSO_4$.

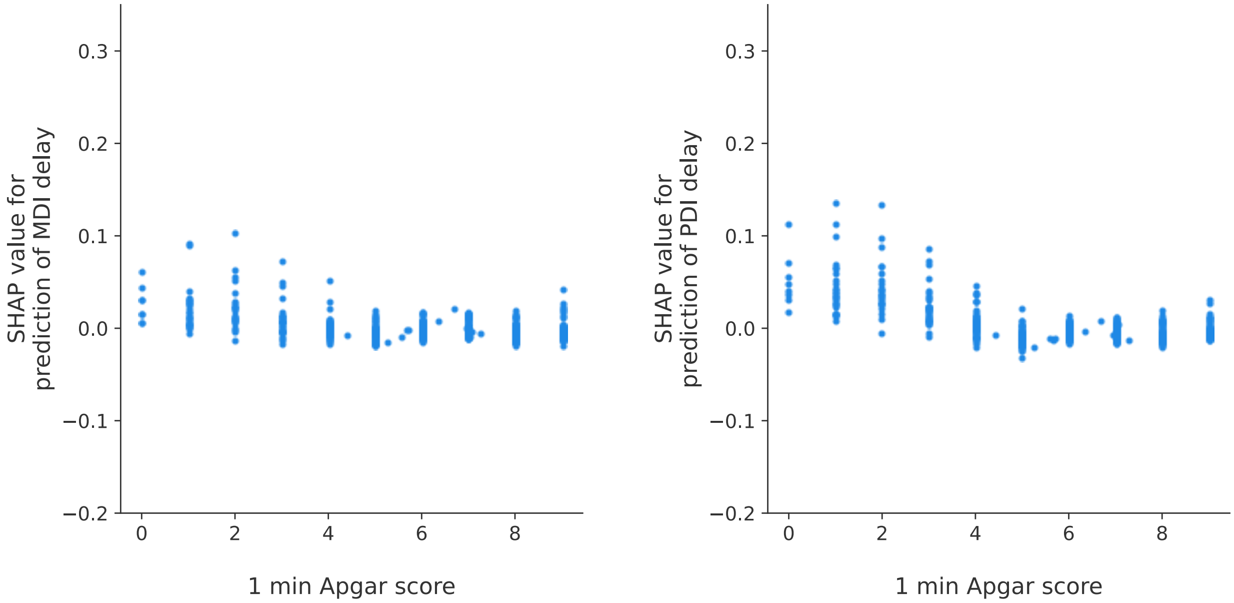

Supplementary Figure 8. The effect of 1 minute Apgar score on mental developmental (MDI) and psychomotor developmental (PDI) delay respectively.

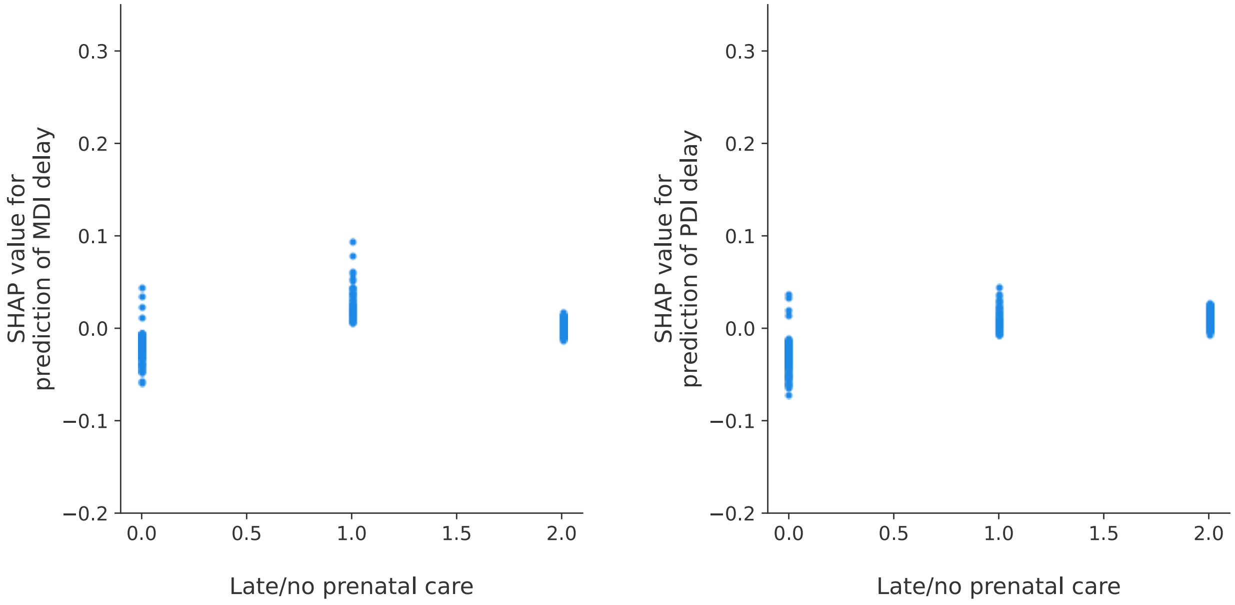

Supplementary Figure 9. The effect of prenatal care on mental developmental (MDI) and psychomotor developmental (PDI) delay respectively. 0 is normal prenatal care

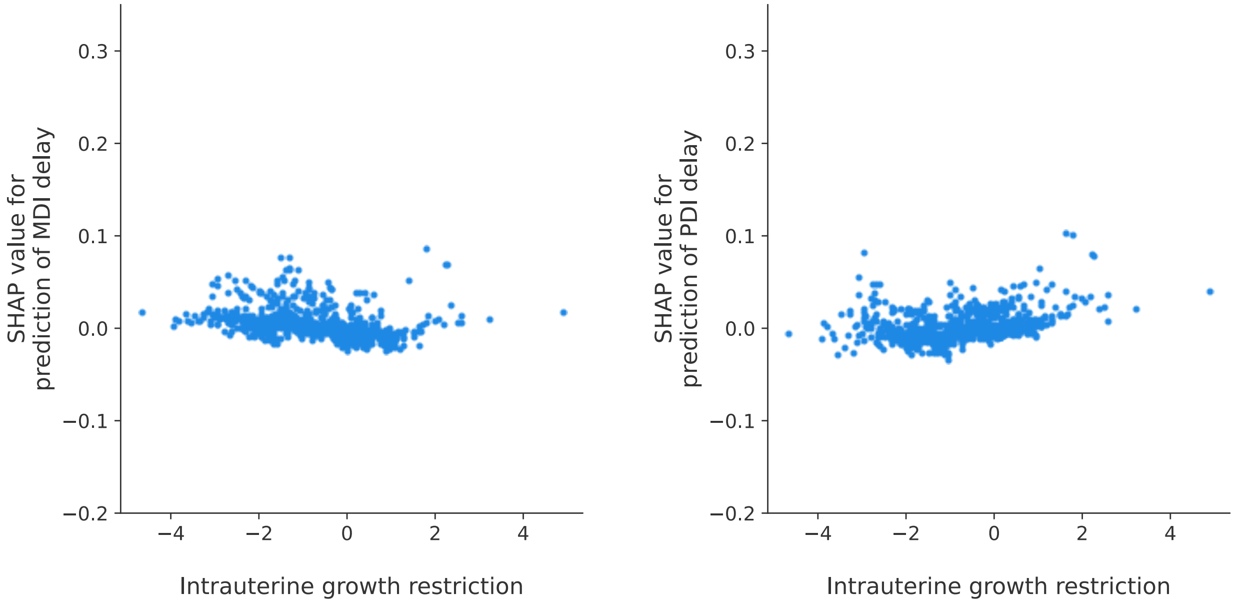

Supplementary Figure 10. The effect of intrauterine growth restriction (IUGR) on mental developmental (MDI) and psychomotor developmental (PDI) delay respectively. Higher values indicate greater IUGR

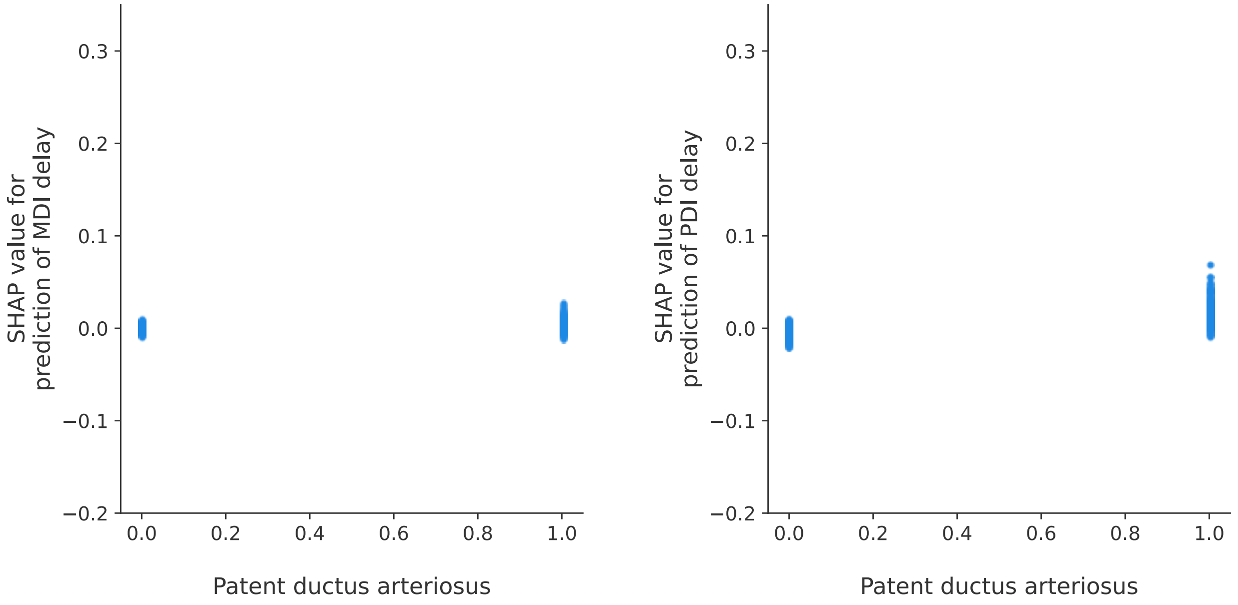

Supplementary Figure 11. The effect of patent ductus arteriosus (PDA) on mental developmental (MDI) and psychomotor developmental (PDI) delay respectively. 0 is no PDA and 1 is PDA

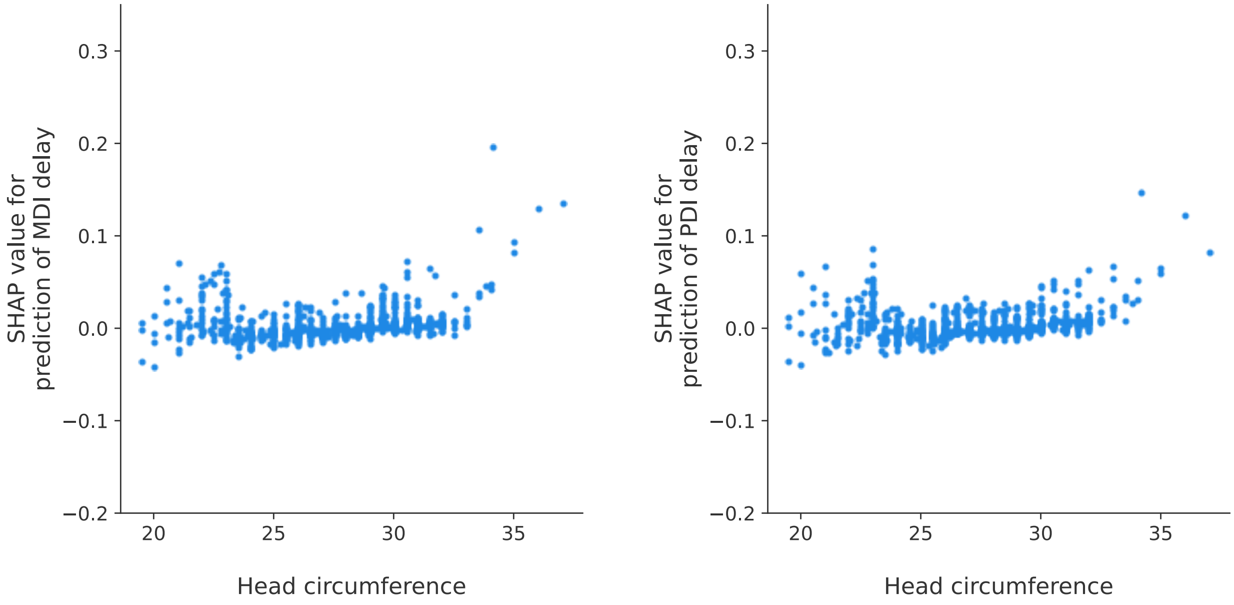

Supplementary Figure 12. The effect of head circumference on mental developmental (MDI) and psychomotor developmental (PDI) delay respectively.

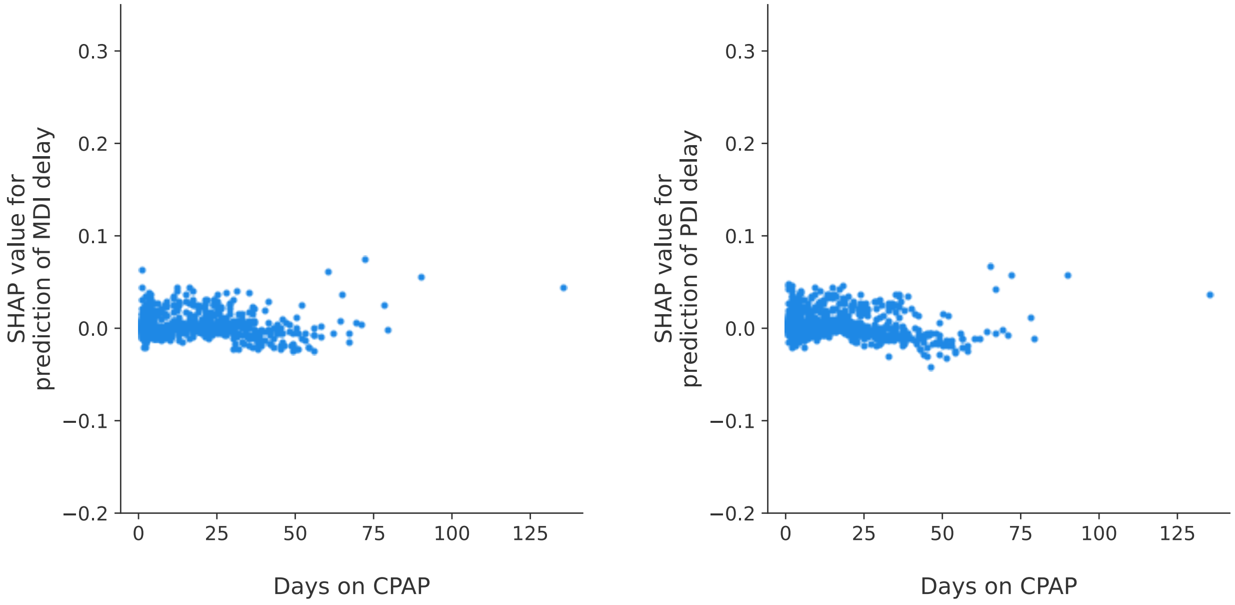

Supplementary Figure 13. The effect of days on CPAP on mental developmental (MDI) and psychomotor developmental (PDI) delay respectively.

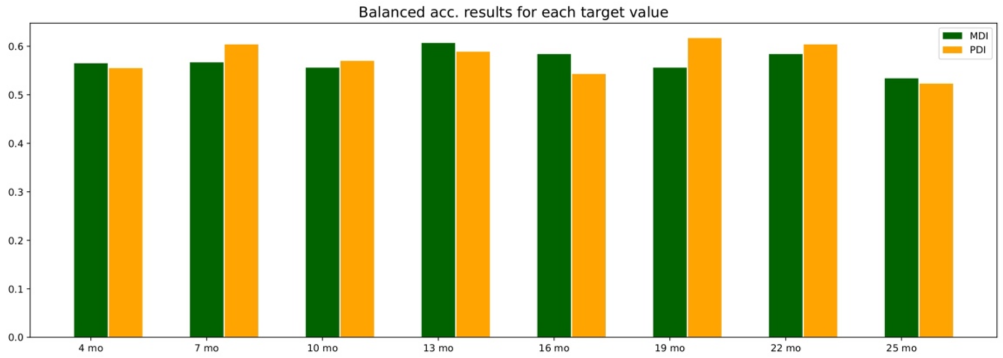

Supplementary Figure 14. Target Value Justification. The figure shows the balanced accuracy results obtained from the Random Forest classification model for various target values on mental and psychomotor developmental delays, with clinical at-birth variables as the fixed predictors.
